# Sialendoscopy and Combined Minimally Invasive Treatment for Large Parotid Stones

**DOI:** 10.1155/2016/1354202

**Published:** 2016-11-02

**Authors:** Jan Rotnágl, Šárka Zavázalová, Olexii Vorobiov, Jaromír Astl

**Affiliations:** Otorhinolaryngology and Maxillofacial Surgery Department of the 3rd Faculty of Medicine of Charles University and the Military University Hospital Prague, Prague, Czech Republic

## Abstract

Sialendoscopy (SE) represents nowadays one of the standard diagnostic and therapeutic procedures in the treatment of major salivary glands lithiasis. We know from experience that it is successful only in small percentage of patients, when used in monotherapy. However, it represents an indispensable part of all of the combined minimally invasive gland-preserving treatment techniques, the success rate of which is around 90%. In this work, we focused on the role of sialendoscopy in the treatment of patients with larger inflamed fixed stones in glandula parotis. We conducted a total of 364 sialendoscopy procedures in 332 patients on our site. We have confirmed lithiasis as a cause of salivary gland obstruction in 246 (74%) patients. In 9 patients there was larger, single, or multiple inflamed fixed lithiasis of glandula parotis. In this subgroup of patients endoscopically assisted sialolithectomy from external mini-incision has become the method of choice. In 9 of the 9 (100%) cases we have achieved complete elimination of stones, and in 8 of the 9 (89%) cases we have achieved complete elimination of complaints. Sialoendoscopically assisted sialolithectomy of glandula parotis from external mini-incision has proved to be highly effective technique to eliminate stones with minimal complications.

## 1. Introduction

Since the 90s of the 20th century, a dynamic development of minimally invasive gland-preserving techniques in the treatment of obstructive major salivary gland diseases is seen, particularly sialolithiasis causing obstruction in up to 80% of cases [1–4]. In this contemporary trend, sialendoscopy plays a pivotal role. While being successful in monotherapy in treatment of lithiasis only in a small percentage of patients [5], however, its combination with other minimally invasive techniques is raising the overall success of gland-preserving treatment that is ranging between 80 and 90%, depending on the affected gland, location and size of the stone, and the technique used [3, 5]. In small, loosen stones in submandibular and parotid glands, the method of the first choice is endoscopic extraction [1, 3–7]. Larger stones whose size does not allow intraductal manipulation are in the case of submandibular gland indicated for intraoral sialolithectomy, which is preferred over ESWL (extracorporeal shockwave lithotripsy) [3, 5, 7–9]. On the contrary, larger stones up to the size of 7 mm located in the parotid gland are preferably solved by ESWL, with lower success rate—about 60%—of complete elimination. Additional 20–30% of patients reach alleviation of symptoms [3, 10–12]. Due to the fact that sialendoscopy is relatively new method, more detailed processing of the long-term effect of gland-preserving treatment is still missing [3]. However, the individual techniques, their limits, and successfulness are already pretty well mapped. Here we quote our result of relatively less traditional technique, endoscopically assisted sialolithectomy from external mini-incision, indication of which applies only to a narrow range of patients [3, 13, 14]. Most of the authors reserve this method for cases of failure of other minimally invasive techniques [3, 4, 12–18]. In our case it involved 9 of the 246 (3.7%) subjects from the total number of patients affected by lithiasis. In the subgroup of patients with lithiasis in glandula parotis, the studied group included 9 of the 32 (28%) subjects.

## 2. Methods

### 2.1. Study Population

In the period from the mid of 2009 to the beginning of 2016 we conducted a total of 364 of sialoendoscopic procedures in 332 patients. We have identified lithiasis as the cause of obstruction in 246 (74%) of patients. Data collection was performed by retrospectively prospective form.

### 2.2. Algorithm of Indication

**Figure 1:**
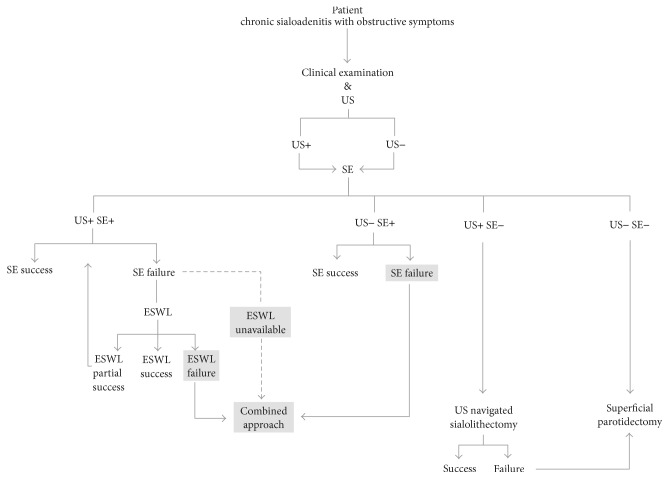
Suggested scheme of diagnostic and therapeutic procedures in patients with clinically manifesting obstructive disease of parotid gland (US: ultrasonography, SE: sialendoscopy, US+/SE+: concrement found, and US−/SE−: concrement not found).

Figure 1 shows a scheme of diagnostic and therapeutic procedures in patients with clinically manifesting obstructive disease of parotid gland. Since we did not have the possibility to use ESWL in our case, the procedure has been simplified. For illustration, we have created the scheme in full.

### 2.3. Instrumentation Used

We had a complete set of semirigid optics available. Diameter of endoscopes was 0.8, 1.1, and 1.6 mm. Resolving power of an image being transmitted was 6,000 pixels. Optics viewing direction was 0°. The field displayed was 70°. Neuromonitoring was NIM-3.0.

### 2.4. Procedure Technique

**Figure 2:**
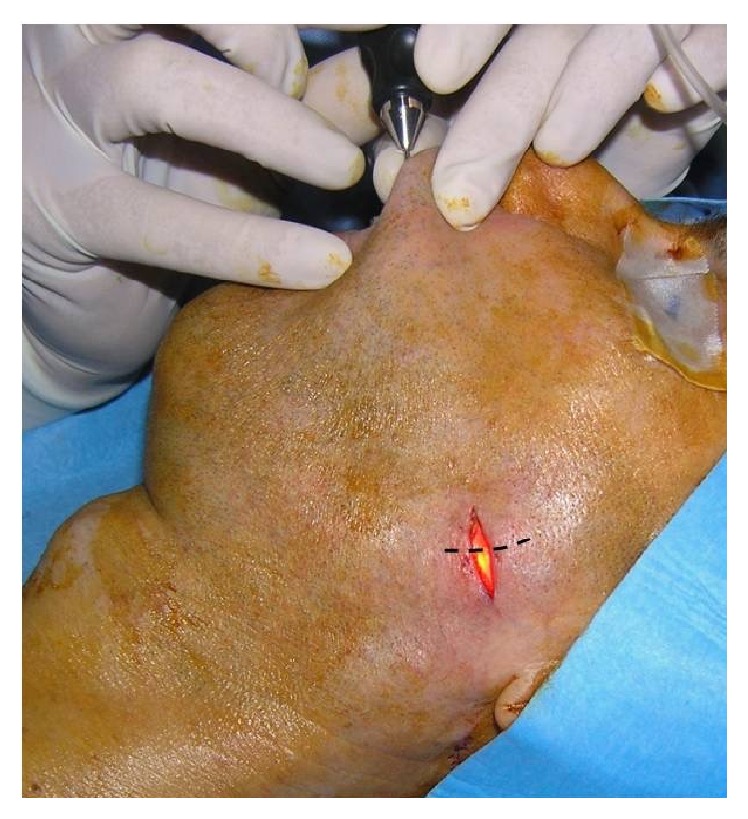
Mini-incision above the light of the endoscope with respect of the main duct direction; dashed line shows recommended incision with respect of relaxed skin tension lines.

**Figure 3:**
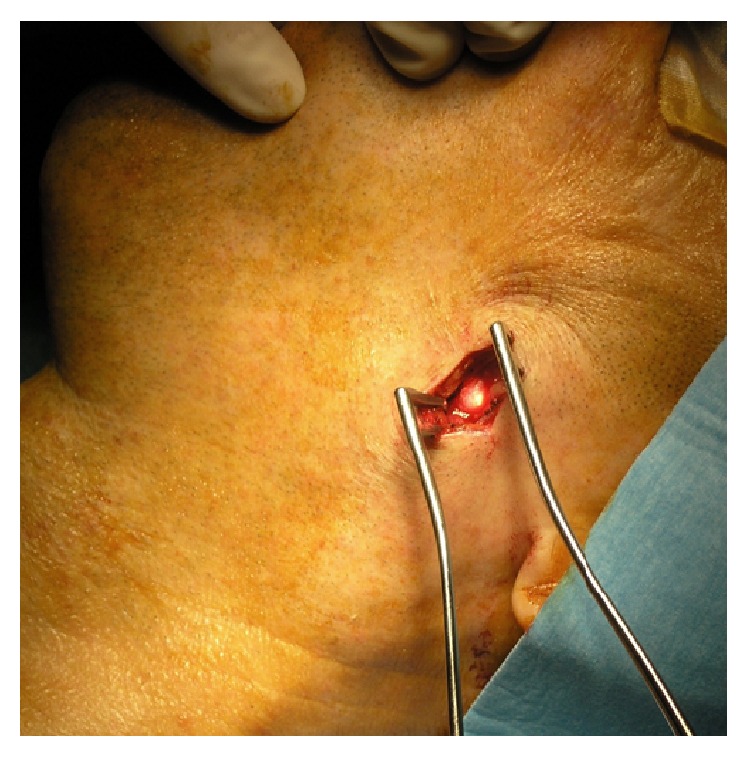
Identification of the duct.

Two patients preferred to undergo the procedure under general anaesthesia; 7 patients were operated on under local anaesthesia. During surgeries under general anaesthesia, we used neuromonitoring n. VII; otherwise the procedure technique was identical in all cases. We have always performed every procedure under antibiotic prophylaxis due to the presence of chronic inflammatory changes of the affected gland. Procedure began with endoscopy, by which we located the front wall of a stone. Then, using transillumination effect we have identified the external location of the stone. Above the light of the endoscope we have performed a mini-incision up to 20 mm long. In the first 4 cases the incision has respected the direction of the main duct. This gave us the possibility to extend the incision and explore the duct broadly if necessary. The extension was not necessary in none of these 4 cases. Therefore in next 5 cases we have respected the relaxed skin tension lines to ensure better cosmetic effect. Then we have gradually, against the light of the endoscope, performed dissection to identify duct (Figures 2 and 3).

**Figure 4:**
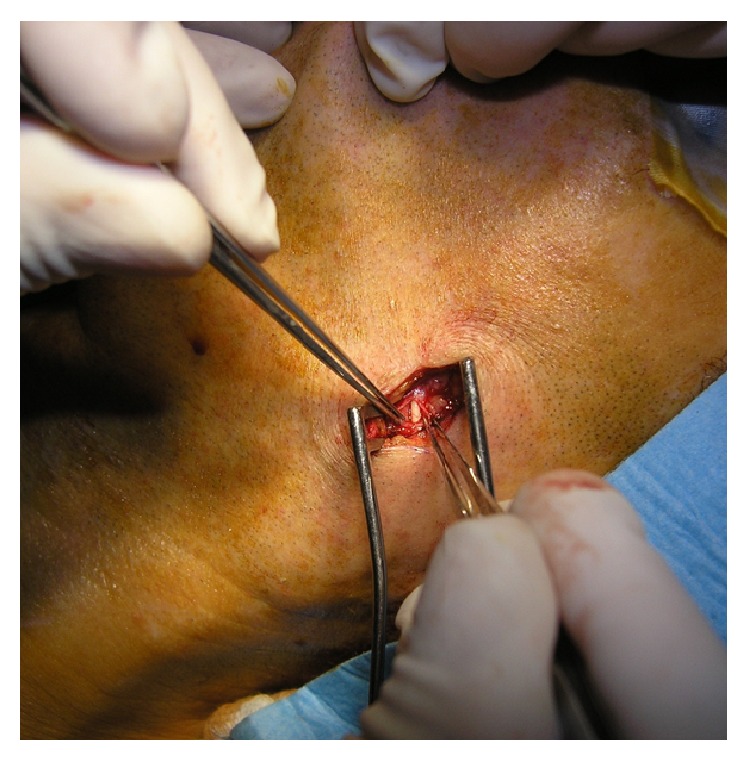
Sialodrain pulled through the area of the split.

By puncture of needle, the tip of which we have endoscopically visualised in front of ventral edge of the stone, we definitely confirmed the correct localization. Subsequently, the stone was removed by splitting the duct. From an external approach, we then introduced endoscope into the proximal part of the split duct in order to eliminate persistent stones or other duplicate pathologies, peripherally from the performed sialolithectomy. Subsequently, again through natural orifice and with use of endoscope, we have introduced, under external control, the intraductal sialodrain, which we pulled through the area of the split to the proximal part of the duct (Figure 4).

The stent was fixed intraorally at the ductal orifice. Externally, we have performed adaptations of the duct and soft tissues over the stent. At the end of the procedure we flushed the stent by corticosteroid solution to prevent stenosis and to check the patency. We have discharged patients the first postoperative day. The stent was left in site for 2-3 weeks; antibiotics were administered for 7 days. We repeated flushing with corticosteroid solution in weeks 1, 2, 4, and 8 after procedure.

## 3. Results

We have identified lithiasis as the cause of obstruction in 246 of the 332 (74%) patients. Lithiasis affected glandula submandibularis in 214 of the 246 (87%) patients and glandula parotis in 32 of the 246 (13%) patients. In 9 of the 32 (28%) patients with affected glandula parotis we have endoscopically identified larger inflammatory fixed lithiasis in the area of proximal third Stensen's duct, or in its primary bifurcation. In 2 cases, the lithiasis was multiple. The average size of stones was 6.8 mm. The group of patients consisted of 7 men and 2 women of average age of 57 years.

In 9 of the 9 (100%) patients we have achieved a complete elimination of stones. In 2 cases we were unable to introduce intraductal stent up to the area of performed ductotomy; therefore the duct was left without stent. In both of these patients, symptoms of obstruction persisted after procedure. In one patient, drainage was restored within 2 months from procedure, with complete remission of complaints. In second patient, obstruction complaints persisted without inflammatory complications even after 2 years after procedure. However, during follow-up endoscopy we have not found any stenosis in the area of previous procedure, and the ductal system was freely accessible without any restrictions for endoscope, with peripheral dilatation of unclear etiology. Mild complaints of the patient have not been yet a reason for radical surgery. Therefore, drainage of affected gland and complete remission of complaints were achieved in 8 out of 9 (89%) patients at follow-up performed in 12 to 36 months after procedure. We have not seen paresis of n. VII in any of the procedures. In 2 of 9 (22%) cases the healing was accompanied by a temporary external secretion of saliva from the wound, which paradoxically ceased almost immediately after removal of the sialostent. In none of the cases we have seen formation of salivary fistula or inflammatory complications of healing. We have not used a traditional S (Blair) incision approach with elevation of dewlap in any of the cases. All 9 sialolithectomies were carried out by mini-incision up to 20 mm (4 times in the direction of duct course, 5 times in the direction of skin fissility), without the need to expand the operational approach.

## 4. Discussion

Sialendoscopy, as part of minimally invasive treatment of stones in major salivary glands, significantly raised the gland-preserving treatment success rate in the past 25 years. With stones in the parotid gland, which do not allow primarily endoscopic removal or mechanical intraductal fragmentation due to their size, individual sites slightly differ in preference of treatment techniques. Larger stones up to 7 mm are usually preferentially solved by ESWL with reported success rate in complete elimination reaching 60%. In another roughly 30% of patients, their clinical complaints are only alleviated. In part of these patients, the partial success of ESWL can be combined retroactively with endoscopic additional removal of residual stone fragments [3, 10, 11]. Recently, there have been also several newer studies published on the use of intraductal laser lithotripsy. Particularly, the use of Thulium-YAG laser in combination with subsequent endoscopic extraction of fragments had been evaluated as relatively safe technique of sialolithectomy. Successfulness in relation with ESWL was comparable in the parotid gland treatment and even twice higher in the submandibular salivary gland treatment [19]. Despite these optimistic results, the long-term reserved attitude towards this technique does not change very much because of existing risks of iatrogenic damage to soft tissues.

Even today, intraductal laser lithotripsy used to treat sialolithiasis does not belong to commonly used methods in the world's leading medical centres [1, 4]. The same applies to the combined sialoendoscopically assisted sialolithectomy of parotid gland which is, due to the need for external incision and relative risk for facial nerve, reserved only for cases of failure of other techniques [3, 4, 12–18].

In our case, this technique became a method of choice in 9 patients. Nationwide unavailability of ultrasound-guided ESWL contributed to it, too. In available references in literature, individual authors also show results for similarly small groups of patients, confirming rarity of the indication. Nahlieli et al. described as one of the first authors the use of combined external approach in 12 patients [14]. He also proposed indication criteria for this procedure as follows: location of the stone in the posterior one-third of Stensen's duct, small duct diameter, and impacted stone larger than 5 mm, and intraparenchymal stones. 9 of the 12 (75%) stones were successfully removed. In 7 of the 12 patients gland functioned normally [14]. McGurk et al. described the use of external approach in 8 patients; from this number, seven patients suffered from lithiasis and one from stenosis. In all 7 cases, the stone was removed successfully. In one case, duct ligature has been performed due to the impossibility to reconstruct the affected duct. Function of the affected gland has been preserved in 75% of patients [13]. Koch et al. described his experience with this technique in 19 patients, 17 of which suffered from lithiasis. The treatment was successful in 89.5% and in 94% in lithiasis, respectively. In two cases it was necessary to perform parotidectomy due to the impossibility to perform duct reconstruction. The conditions of successful treatment achievement were the possibility to reach the pathology endoscopically, the possibility to reconstruct the duct, and the ability to recover the function of the affected gland [17]. Marchal described 37 patients with refractory stones larger than 6 mm, and with duct stenosis. The treatment was successful in 92% of patients [20]. Konstantinidis et al. described similar experiences in 12 patients with lithiasis. Sialolithectomy has been successful in 100% of cases. Intraductal stent was used in 9 of the 12 patients. The follow-up endoscopy discovered mild stenosis without clinical manifestation in 7 of the 12 cases; the follow-up scintigraphy showed normal function in 11 of the 12 cases [18]. Mikolajczak et al. [16] and Kopeć et al. [12] come up with similar results. On the contrary, for example, Numminen et al. report successful combined treatment in 6 of the 8 patients without the use of stent [15].

Upon failure of previous therapy, or in unavailability of ESWL, this method can be the only treatment option before radical resection. Understandable condition for success is the endoscopic accessibility of ventral edge of the stone [17]. In our group, all of the 9 patients were affected by lithiasis which meets this condition. Stones were successfully removed in 9 of the 9 (100%) cases, and the physiological function of the gland was restored in 8 of the 9 (89%) patients. In one patient, mild problems with obstruction persisted but have not required radical treatment. The follow-up endoscopy, however, has not proved stenosis in site of the surgery. From our experience, stenting of the duct after the performed surgery did not appear essential for the preservation of duct patency. Nevertheless, good long-term results in 7 of the 9 patients, who had the postoperative stent, allow us to rather recommend its use. But we have also noticed probable obstructive action of the stent for peripheral part of gland that resulted in external secretion from the wound in 2 cases. Removal of the stent and compression resulted, in both of the cases, in almost immediate cessation of the external secretion. From the long-term perspective, this fact had no effect on the overall favourable healing and physiological function of the gland. Endoscopically assisted sialolithectomy can be performed under general and/or local anaesthesia, depending on the surgical approach. Majority of procedures referred to in literature are performed under general anaesthesia [12, 14–18]. In all cases of our group, mini-incision proved to be sufficient surgical approach, allowing the procedure to be performed under a local anaesthesia. General anaesthesia provides the surgeon with greater convenience and possibility to use neuromonitoring. On the contrary, performing the procedure under local anaesthesia makes this procedure more acceptable in the eyes of patients, and less serious, and it can be performed as an ambulatory procedure. Since in our group we have not noticed any complication resulting from the use of local anaesthesia, in our view it can be recommended for well-selected patients.

## 5. Conclusion

Sialoendoscopically assisted glandula parotis sialolithectomy from external mini-incision proved to be highly successful technique to eliminate stones with minimal complications. In larger fixed stones in glandula parotis, refractory to other therapies, and/or in unavailability of ESWL, this technique can be recommended as a suitable alternative to minimally invasive treatment that saves the patient from radical resection. The only condition for success is the ability to reach the stone with endoscope.

Our results show that, in properly selected patients, this procedure is very well tolerated even under local anaesthesia, which further increases its attractiveness.
